# Retraction statement: MicroRNA‐520c enhances cell proliferation, migration, and invasion by suppressing IRF2 in gastric cancer

**DOI:** 10.1002/2211-5463.13511

**Published:** 2022-11-15

https://doi.org/10.1002/2211-5463.12142

The above article published online on 13 October 2016 in Wiley Online Library (wileyonlinelibrary.com), has been retracted by agreement between the Editor‐in‐Chief, John Wiley and Sons Ltd and the authors. The retraction has been agreed following an investigation based on allegations raised by a third party, which revealed inappropriate and unexplained duplications between this and another article [1]. Thus, the editors consider the conclusions of this manuscript substantially compromised.
